# HIV-1 Infection of T Cells and Macrophages Are Differentially Modulated by Virion-Associated Hck: A Nef-Dependent Phenomenon

**DOI:** 10.3390/v5092235

**Published:** 2013-09-18

**Authors:** Alyssa Cornall, Johnson Mak, Alison Greenway, Gilda Tachedjian

**Affiliations:** 1 Centre for Biomedical Research, Macfarlane Burnet Institute for Medical Research and Public Health, Melbourne 3004, Victoria, Australia; E-Mails: alyssa.cornall@mcri.edu.au (A.C.); greenway@burnet.edu.au (A.G.); 2 Regional HPV Labnet Reference Laboratory, Department of Microbiology and Infectious Diseases, The Royal Women’s Hospital, Parkville 3052, Victoria, Australia; 3 Murdoch Children’s Research Institute, Parkville 3052, Victoria, Australia; 4 School of Medicine, Faculty of Health, Deakin University, Geelong 3220, Victoria, Australia; E-Mail: johnson.mak@deakin.edu.au; 5 Commonwealth Scientific and Industrial Research Organisation, Livestock Industries, Australian Animal Health Laboratory, Geelong 3220, Victoria, Australia; 6 Department of Microbiology, Monash University, Clayton 3168, Victoria, Australia; 7 Department of Infectious Diseases, Monash University, Melbourne 3004, Victoria, Australia

**Keywords:** HIV-1, Nef, Hck, macrophage, T lymphocyte, virion, infectivity

## Abstract

The proline repeat motif (PxxP) of Nef is required for interaction with the SH3 domains of macrophage-specific Src kinase Hck. However, the implication of this interaction for viral replication and infectivity in macrophages and T lymphocytes remains unclear. Experiments in HIV-1 infected macrophages confirmed the presence of a Nef:Hck complex which was dependent on the Nef proline repeat motif. The proline repeat motif of Nef also enhanced both HIV-1 infection and replication in macrophages, and was required for incorporation of Hck into viral particles. Unexpectedly, wild-type Hck inhibited infection of macrophages, but Hck was shown to enhance infection of primary T lymphocytes. These results indicate that the interaction between Nef and Hck is important for Nef-dependent modulation of viral infectivity. Hck-dependent enhancement of HIV-1 infection of T cells suggests that Nef-Hck interaction may contribute to the spread of HIV-1 infection from macrophages to T cells by modulating events in the producer cell, virion and target cell.

## 1. Introduction

The human immunodeficiency virus type 1 (HIV-1) accessory protein Nef enhances both viral replication and infectivity in primary target cells and cell lines [1,2,3,4,5,6,7,8,9,10]. This may be facilitated by several well-characterised functions of Nef which include modulation of cell receptor expression [8,11,12,13,14], cell activation pathways [15,16], localization of cellular proteins and cholesterol [17,18,19], and apoptosis pathways [20,21,22]. Nef is also present in cell-free viral particles [23,24], and several studies demonstrate that the presence of Nef during virion production enhances viral infectivity [24,25,26]. Some host cell proteins reported to interact with Nef, including MAPK and Lck, are also found within the virion [27,28].

As Nef is a non-structural protein and has no enzymatic activity, it mediates its effects by manipulating the function and/or localisation of host cell proteins [29,30]. The proline repeat motif (PxxP) of Nef has been identified as a binding motif for many of its binding partners, and is important for Nef function [29,31,32,33,34]. In particular, the SH3 domain of cellular proteins is known to bind strongly to this motif [4,8,35,36,37,38,39,40]. The SH3 domain of the Src kinase, Hck, binds the PxxP motif of Nef with affinity and specificity approximately 1-2 orders of magnitude higher than that observed for other SH3-ligand interactions [34,40]. This interaction results in upregulation of kinase activity in cell-based and cell-free assays [34,41,42,43,44], suggesting that Nef may mediate some of its effects by modulating the activity of these kinases during infection. The SH3 domain of the macrophage-specific Src kinase, Hck, binds the PxxP motif more strongly than other SH3-ligand interactions [34,40]. Several studies indicate a role for Hck in enhancing viral infection of HeLa cells and T cell lines [45]. Whether this enhancement of infection is dependent on Nef has not previously been reported. It is also unclear whether Hck, like Lck, is incorporated into viral particles, or whether virion incorporation of host cell proteins is dependent upon interaction with Nef.

The role of Hck during HIV-1 infection is particularly significant, as macrophages play a distinct role during the spread of infection and disease in infected individuals. They are important for the spread of virus from the circulatory system to tissue, including into the brain [46], and facilitate infection of other cell types including lymphocytes [47,48,49]. Macrophages contribute to HIV-1 persistence by forming reservoirs [50,51]. Nef and Hck have both, individually or together, been implicated in many of these events, either directly or by their involvement in relevant signaling pathways [49,52,53,54,55], making them potential drug targets to reduce viral spread. Recently, attention has turned to cellular proteins as potential drug targets, and an increasing number of studies are identifying candidate drug classes with potential to inhibit Nef- and Hck-mediated enhancement of viral replication [56,57,58,59]. One drug class in particular, diphenylfuropyrimidines (DFPs), selectively inhibits Nef-dependent activation of Src-family kinases and also inhibits HIV replication [56,59].

In this study we determined whether Hck interacts with Nef during HIV-1 infection of macrophages and the effect this interaction may have on virus production in infected macrophages. We also examined whether the interaction affected incorporation of Hck into the virion, and the subsequent impact that virion-associated Hck had on the efficiency of HIV infection of primary monocyte-derived macrophages and primary T cells.

## 2. Results and Discussion

### 2.1. Nef Co-Precipitates Endogenous Hck from HIV-1 Infected Macrophages

**Figure 1:**
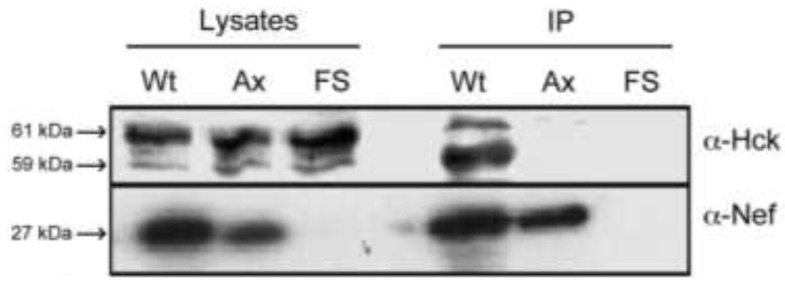
Co-precipitation of Hck from HIV-1 infected macrophages by Nef. Lysates of primary Monocyte-Derived Macrophages (MDM) infected with AD8 (Wt), AD8AxxA (Ax) or AD8FSNef (FS) were immunoprecipitated with sheep anti-Nef sera. Immunoblots of cellular lysates and precipitates were probed with anti-Nef and anti-Hck monoclonal antisera. Data are representative of 3 independent assays.

Nef interacts strongly with the SH3 domain of Hck, via its proline repeat motif, both in cell independent and cell-line based assays [36,37,41,42,43,59]. However, this interaction has not yet been confirmed during HIV-1 infection of primary cells. In order to confirm that Nef interacts with endogenous Hck during HIV-1 infection of primary macrophages, lysates from primary monocyte-derived macrophages infected with HIV-1 AD8, AD8AxxA (expressing a proline repeat mutant form of Nef) or AD8FSNef (Nef-deleted mutant) were subjected to immunoprecipitation with Nef antibody, and the co-precipitated proteins were separated by SDS-PAGE and probed with Hck specific antibodies. Nef was observed to co-immunoprecipitate Hck from infected macrophages, and this interaction was dependent on the presence of an intact proline repeat motif in Nef (Figure 1). In whole cellular lysates, at least three forms of Hck, consistent with macrophage-expressed forms of Hck, were observed, which likely correspond to the non-palmitoylated and palmitoylated forms of p59, as well as p61 Hck, and may also include differentially phosphorylated forms of Hck [60,61,62,63]. The 61 kDa form appeared to be the most abundant in macrophage lysates. The 59 kDa form of Hck appeared to interact most strongly with Nef, followed by the 61 kDa form. This may be due to the fact that both Nef and p59 Hck associate predominantly with the plasma membrane, while p61 Hck associates predominantly with the cytoplasmic face of lysosomal membranes [60,61,62,63]. The Nef AxxA mutant protein was expressed at slightly lower levels than the wild-type Nef protein; however substantial amounts of both Nef and Nef AxxA proteins were immunoprecipitated by the anti-Nef antibody. These data show that Nef co-precipitates endogenous Hck in HIV-1 infected Monocyte-Derived Macrophages (MDMs) and that the interaction is mediated through the Nef proline repeat motif.

The interaction between Nef and Hck during HIV-1 infection of macrophages has important implications for the progression of infection. Nef has been implicated in many events important for macrophage-mediated spread of HIV-1 infection, including secretion of inflammatory molecules, which recruit leukocytes to the site of infection and make them more susceptible to infection [48,49,53,54]. Nef also enhances viral replication in primary macrophages, by mechanisms that remain unclear [9,49,64]. This makes Nef and the Nef-Hck interaction a potential drug target to reduce viral spread and HIV-1 associated pathogenesis [59]. The results of the present study confirm that this interaction occurs in HIV-1 infected macrophages.

### 2.2. Nef and the Nef PxxP Motif Enhance HIV-1 Replication and Infectivity in Primary Monocyte-Derived Macrophages (MDMs)

**Figure 2:**
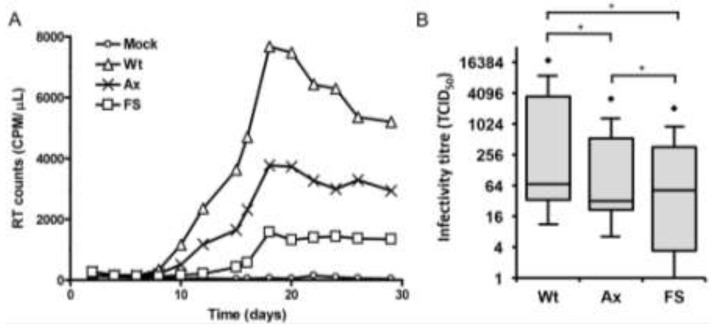
The effect of Nef and Nef PxxP on HIV-1 replication kinetics and infectivity in primary MDM. (**A**) HIV-1 replication kinetics in primary MDM. Cells were infected with equivalent amounts of AD8 (Wt; triangles), AD8AxxA (Ax; crosses) or AD8FSNef (FS; squares) based on cell-free RT activity. The results presented are from a single donor and are representative of experiments conducted in cells from 8 separate donors; (**B**) HIV-1 infectivity in primary MDM. Cells were infected with equivalent amounts of AD8 (Wt), AD8AxxA (Ax) or AD8FSNef (FS) based on cell-free RT activity. The infectivity titreof each virus was measured by TCID_50_ assay in primary MDMs fromeachofseven donors. Significant differences in infectivity titres (*p* < 0.001; Wilcoxon signed-rank test) are indicated with as asterisk (*). Outliers are indicated with diamond shapes.

Nef enhances viral replication in primary T cells and increases viral infectivity 4- to 40-fold in T cell lines [5,65] and HeLa-derived indicator cell lines [2,6,9,32,33]. To determine the role of the Nef proline repeat motif in the replication of HIV-1 in MDMs, we compared the replication of the wild-type AD8 with AD8AxxA and AD8FSNef. HIV-1 stocks were generated by transfection of 293T cells that do not express Hck. Equivalent virus normalized to virion-associated reverse-transcriptase (RT) activity was used to infect MDMs. Since HIV-1 infection of MDMs was performed with a relatively high MOI and these cells become chronically infected, the data predominantly reflects virus produced from a single round of infection. We did not use inhibitors to block subsequent rounds of virus infection since a previous study, using similar conditions, has demonstrated little difference between HIV-1 production in MDMs in the absence and presence of soluble CD4 used to block new infections [66]. HIV-1 replication in MDMs was relatively slow, with detectable amounts of virus not observed in the supernatant until approximately day 10 post-infection, reaching a peak between days 18 and 20 post-infection. In most experiments the peak of HIV-1 replication was not delayed in either AD8AxxA or AD8FSNef. HIV-1 production remained relatively stable after the peak was reached, and significant amounts of virus were still observed in the culture media 30 days post-infection except for wild-type where virus production started to decline, likely due to a decrease in tat mRNA and Tat activity in MDMs as previously described [66]. In experiments conducted in cells from 8 different donors, AD8 produced approximately 2-fold more virus than AD8AxxA and up to 8-fold more virus at the peak of infection (between days 18 and 20 post-infection) than AD8FSNef (Figure 2A). We next examined the role of Nef and the Nef proline repeat motif in the enhancement of HIV-1 infectivity in primary MDMs as determined in a tissue culture infectious dose 50 (TCID_50_) assay. The infectivity titres of AD8 varied by up to approximately 100-fold between cells derived from seven different donors, indicating significant donor variability (Figure 2B). Mutation of the proline repeat motif had a significant (*p* < 0.001) negative effect on viral infectivity in primary MDMs, reducing infectivity by approximately 50%. The infectivity titre for Nef-negative virus varied by up to 1,000-fold between donors, indicating donor-dependent variations in the requirement for Nef, but overall was significantly lower than both wild-type and Nef proline repeat mutant viruses (*p* < 0.001).

These data confirm the importance of Nef in enhancing HIV-1 replication and infectivity in MDMs and indicate that the proline repeat motif has a role in mediating this effect. This result suggests that Nef interaction with the SH3 domain of one or more signaling proteins, such as Src kinases, may be important for viral replication and infectivity in macrophages. Inhibitors of Nef-dependent Src kinase activation have been used to demonstrate that activation of Src kinases by Nef enhances virus production in cell lines [56,59]. The results of those studies indicate that Nef-dependent enhancement of virus production is largely dependent on Nef-Src kinase interactions in the cell lines used. Our results confirm that the PxxP motif of Nef plays a role in enhancing viral replication and infectivity in primary macrophages. Since we have shown that Nef interacts with Hck in MDMs, the phenotype observed could be due in part to this interaction in the target cell. In this regard, the upregulation of Hck tyrosine kinase activity as a consequence of interaction with Nef has been demonstrated consistently in cell lines and cell-independent assays [37,41,42,59,67,68]. However, the additional impairment of replication observed in the presence of the deltaNef mutant clearly indicates that interaction with Hck, or other SH3-domain containing cellular proteins, is not the only mechanism by which Nef enhances viral replication and infectivity in primary macrophages. Nef has multiple cellular binding partners, with which it interacts via other motifs in addition to the PxxP motif, and which have also been implicated in enhancement of viral replication (reviewed in [29]).

### 2.3. Nef and Hck Incorporation into HIV-1 AD8 Virions

**Figure 3:**
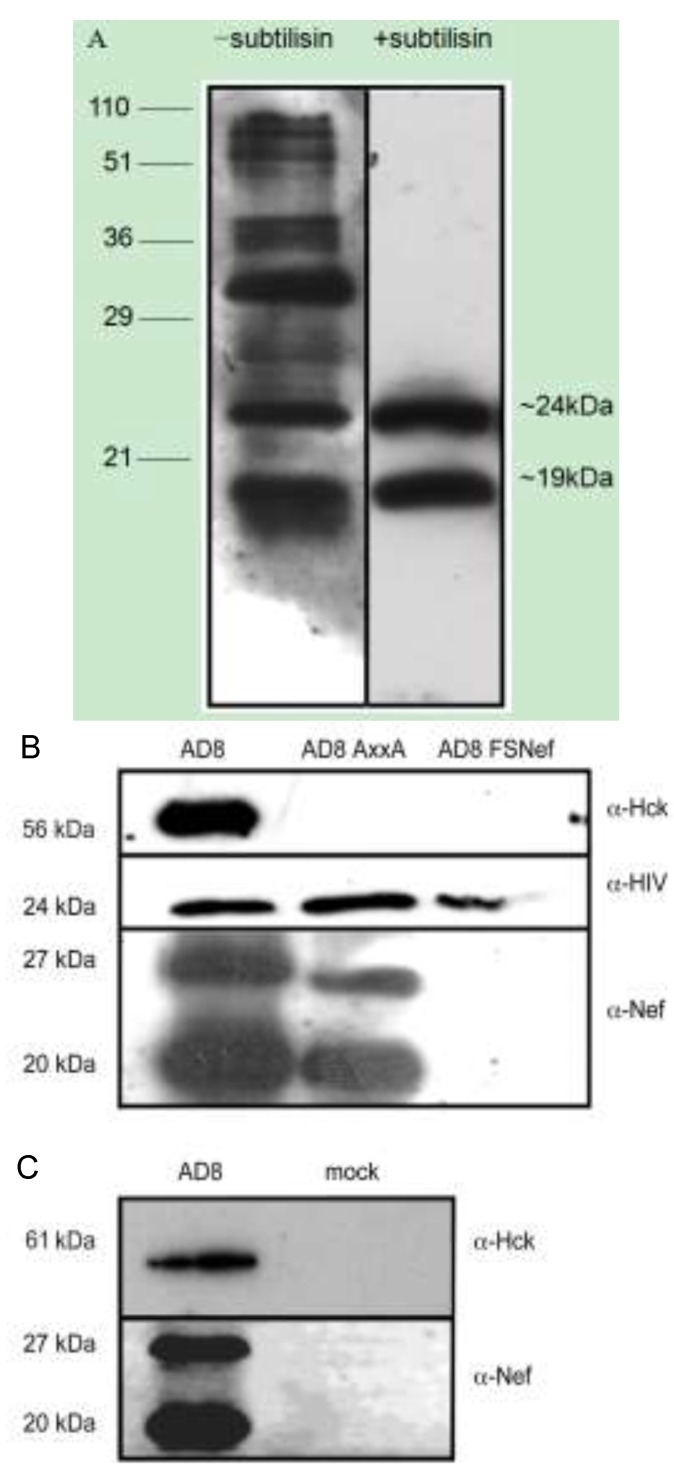
Nef and Hck are present in HIV-1 virions derived from 293T cell lines and primary monocyte-derived macrophages. (**A**) Immunoblots of lysates of pelleted viral material generated by transfection of 293T cells with pAD8-1, with and without subtilisin digestion, probed with HIV antisera. The molecular weights of standard markers are indicated in kDa on the left-hand side of the figure. The molecular weights on the right hand side of the figure represent the approximate size of two major reactive products which likely correspond to viral capsid and nucleocapsid proteins; (**B**) HIV-1 viral particles purified by subtilisin treatment of pelleted virus isolated from the supernatants of 293Ts co-transfected with pAD8-1, pAD8AxxA or pAD8FSNef and pHck; or (**C**) of monocyte-derived macrophages infected with AD8 or mock-infected; were subjected to SDS-PAGE and probed with anti-Hck and EH1 anti-Nef mouse monoclonal sera. Data are representative from 2 independent experiments for each cell type.

Our data show that Nef interacts with Hck in HIV-infected cells, in a manner dependent on the proline repeat motif, and that this same motif of Nef contributes to viral replication and infection of HIV-1 in infected primary macrophages. Nef-dependent enhancement of infectivity is partly dependent on its presence in the target cell, and partly dependent on its presence in the virion [1,3,4,5,6,9,10]. Cellular kinases such as Lck and MAPK are incorporated into the virion and this may have an effect on infectivity [27,28]. Hck may therefore also be present in the virion, where it may have an effect on infectivity; and, given the strength of the Nef-Hck interaction, it is also possible that Nef may mediate virion incorporation of Hck. To examine this hypothesis, HIV-1 derived from 293T cells transfected with pAD8-1, pAD8AxxA or pAD8FSNef, and pHck (which encodes murine Hck), were concentrated by centrifugation through a sucrose cushion followed by subtilisin digestion to purify viral particles by removing host cell-derived microvesicles. In Figure 3A, pelleted viral supernatant from transfected 293T cells with and without subtilisin digestion were immunoblotted with anti-HIV sera. In the subtilisin-treated lysates, only two major reactive proteins were detected (matching the expected sizes of capsid and nucleocapsid), in contrast to the un-treated lysates. This indicates that the subtilisin digestion has degraded the outer membrane and associated proteins of the viral particles. Immunoblots of subtilisin-purified viral lysates were probed with anti-Nef, anti-Hck and anti-HIV sera, shown in Figure 3B. Two bands, approximately 20 kDa and 27 kDa in size, were observed when AD8 viral lysates were probed with anti-Nef. These bands correspond to the cleaved and intact forms of Nef, respectively. The 20 kDa band was of greater intensity than the 27 kDa band, as expected from earlier studies [24,69]. The same two bands corresponding to Nef (20 kDa and 27 kDa) were also observed in AD8AxxA viral lysates, although these bands were of decreased intensity in comparison to those seen in AD8 viral lysates. A strong band reactive to Hck antibodies, of approximately 56 kDa, consistent with the size of murine p56 Hck, was also observed in AD8 virion preparations. No band corresponding to Hck was observed in preparations of AD8AxxA or AD8FSNef, indicating that incorporation of p56 Hck into 293T-derived virions is dependent on the proline repeat motif of Nef. 

To test for the presence of Nef and Hck in macrophage-derived virions, supernatant from HIV-1 infected cells derived from multiple donors were pooled, and pelleted viral cores purified by subtilisin treatment. Preparations of AD8, which were probed with anti-Nef contained two bands of approximately 20 kDa and 27 kDa, corresponding to the cleaved and full-length forms of Nef, respectively (Figure 3C). The 20 kDa band was more intense than the 27 kDa band as expected. A narrow band reactive to anti-Hck, with a calculated molecular weight of approximately 61 kDa, was also observed in macrophage-derived AD8 viral particles. It is interesting that only one form of Hck was incorporated into macrophage-derived viral particles, and is not simply because membrane-associated Hck was removed when the virions were treated with subtilisin, as the same result was observed in virions which were not digested with subtilisin (data not shown). A recent study has shown that Nef activation of the p61 isoform, and not the p59 isoform of Hck leads to cell fusion when macrophages are infected with HIV [55]. Due to impaired levels of replication, insufficient virus was generated by infection of MDMs with AD8AxxA and AD8FSNef to confirm this result in the absence of wild-type Nef, so it is unclear as to whether Hck is incorporated into MDM-derived virions in the absence of Nef. No contaminating bands corresponding to either Nef or Hck were observed in purified supernatant from mock-infected MDM cultures.

We have observed the Nef-dependent incorporation of Hck into viral particles; specifically, incorporation was dependent on the proline repeat motif of Nef. The detection of Hck in subtilisin-purified virions suggests that it is specifically transported into the virion, rather than simply associating with the viral envelope as the HIV-1 virion buds through the plasma membrane [70]; or that it binds other molecules which localise to the viral core. Specific localisation to the core may suggest that Hck has a function in the virion; this may either be orchestrated by the virus in order to enhance the viral life-cycle, or it may be a defense mechanism instigated by the cell in order to inhibit viral infection and/or replication. Infection events mediated by core components or that involve the viral core include cytoplasmic delivery of the core, core disassembly, formation of the pre-integration complex, reverse transcription and integration (reviewed in [71,72,73]).

### 2.4. Hck Inhibits HIV-1 Infection of Monocyte-Derived Macrophages and Enhances Infection of Primary Lymphocytes

**Figure 4:**
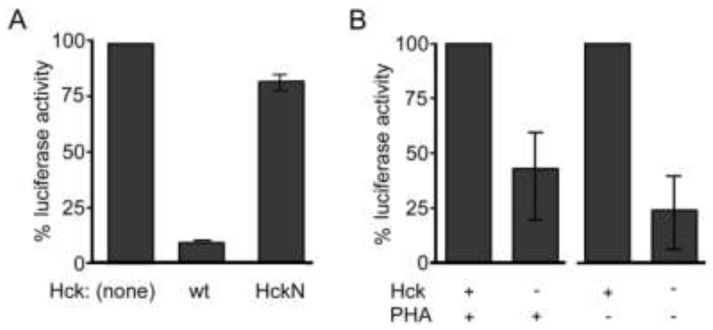
Hck activity inhibits the infectivity of HIV-1 in primary MDMs but not peripheral blood lymphocytes (PBL). (**A**) HIV-1 luciferase reporter viruses expressing AD8 envelope were generated in the presence of expression vectors for wild-type Hck or a kinase-negative mutant of Hck. Primary MDMs were infected with these reporter viruses and the relative infectivity of each was assessed. The results presented are the means from duplicate experiments conducted in cells isolated from one donor and are representative of results observed in cells isolated from three donors; (**B**) HIV-1 luciferase reporter viruses expressing NL4-3 envelope were generated in the presence and absence of an expression vector for Hck. Primary PBL were infected with these reporter viruses either 3 days prior to (PHA−) or on the day of (PHA+) PHA stimulation. The relative infectivity of each virus was assessed and the results presented are the means from experiments conducted in three separate experiments and donors. Error bars indicate standard error.

We have shown that Nef facilitates the incorporation of Hck into virions. In order to determine the effect of Hck activity on HIV-1 infection of monocyte-derived macrophages, HIV-1 luciferase reporter viruses expressing envelope protein from HIV-1 AD8 were generated in the presence of wild-type and dominant kinase-inactive Hck (HckN). This experiment was conducted in the presence of Nef as results from earlier experiments indicate that Nef is required for incorporation of Hck into 293T-derived virions. Due to donor variability [74], representative data generated in MDMs from a single donor have been presented in Figure 4A. In comparison to control virus, the infectivity of HIV-1 generated in the presence of Hck was reduced by up to 90% (Figure 4A) in macrophages from three donors. In the presence of a truncated, dominant kinase-negative form of Hck, infectivity was restored to near or above control levels. This result differs from data demonstrating that the PxxP motif of Nef, which is required for Nef-dependent enhancement of Hck activity, enhances viral replication and infection of macrophages. It suggests that Hck plays distinct roles in the producer cell and the target cell. It also indicates that Hck may be responsible for phosphorylation events of viral proteins that inhibit rather than enhance infection of macrophages. Hck is known to inhibit infection, in the absence of viral protein Vif, by phosphorylating, and therefore facilitating virion incorporation of the inhibitory factor APOBEC3G [75]; however all viral clones used in this study expressed Vif.

In contrast to the results seen in macrophages, previous studies using a dominant kinase-negative Hck have shown that Hck activity enhances infectivity of T cell lines [45]. To determine whether the presence of wild-type Hck would enhance infectivity of primary T cells, peripheral blood lymphocytes (PBL) from three donors were infected in separate experiments with HIV-1 luciferase reporter viruses generated in the presence and absence of wild-type Hck (Figure 4B). Cells from each donor were infected 3 days prior to, or 5 days after, activation with PHA and IL-2. In the absence of Hck, a 60% and 75% decrease in infectivity compared to controls was observed both in cells stimulated prior to infection (PHA+) and in cells activated after infection (PHA-), respectively. These results demonstrate that Hck enhances HIV-1 infection of primary T cells, independently of activation state, suggesting that the observed effect is not due to modulation of cell activation or gene transcription by Hck. It is unclear how Hck may enhance infection of T cells. One study has suggested that Hck may enhance fusion between the viral and cellular membranes based on the evidence that Hck and Lck increase syncytia formation by HIV-1 [45]. Hck may also modify components of the virion. Several viral components are required to be phosphorylated in order to facilitate infection [76,77]. It is unclear as yet whether Hck may enhance viral infectivity by phosphorylating viral proteins. However these results suggest that Hck may facilitate spread of HIV from macrophages to T lymphocytes, by modifying virions in a manner that specifically targets infection of T lymphocytes.

We were unable to establish whether Nef modulates the infectivity of macrophage-derived HIV-1 used to infect primary T cells. Preliminary experiments resulted in inconclusive results, which were heavily influenced by donor variability, predominantly variability in the donor macrophages used to generate the virus (data not shown). This area requires further research to better characterise the effect of Nef-Hck interaction on viral spread.

## 3. Experimental

### 3.1. HIV-1 Molecular Clones and Reporter Constructs

The CCR5-tropic HIV-1 laboratory-adapted molecular clones pAD8-1 and pAD8FSNef have been previously described [78]. The pAD8FSNef clone contains a premature termination sequence in the *nef* gene, resulting in a deltaNef phenotype. The Nef proline repeat motif mutant clone, pAD8AxxA, was generated by mutation of the proline coding sequences (P69, P72, P75 and P78) by PCR site-directed mutagenesis to encode alanine residues using primers 5' Proline mutant [GGT GGG TTT GCC AGT CAG AGC TCA GGT AGC TTT AAG AGC AAT GAC TTA CAA] and the reverse complement of this primer, 3' Proline mutant (Genset, Paris, France). The nucleotide sequence of the mutated product was confirmed by Sanger sequencing using primers Nef5' [ATA TAA GAA TGC GGC CGC CAC CAT GGG TGG CAA GTG GTG AAA A] (Genset) and AD8 Nef3' [CGT CTA GAT CAG TCT TTG TAG TAC TCC GG] (Beckman Coulter, Gladesville, Australia). The luciferase reporter construct pNL4-3.Luc.Rev-R-E- [79] and HIV-1 envelope expression vectors pNLA1 [80] and pNLA1-AD8env [81] have been described. The Nef-deleted expression vector pNLA1Nef (−) was generated by PCR site directed mutagenesis, in which an early termination codon was introduced in the Nef coding gene immediately after the 4th Nef coding amino acid. This resulted in a truncated version of 4 amino acids peptide expression rather than the full length Nef protein. Restriction endonuclease BamHI and XhoI were used to facilitate the cloning. Src kinase expression vector pHck, expressing murine p56 Hck, was as published [82]. The kinetics of interaction between Nef and murine Hck have been shown to be consistent with those between Nef and human Hck [67]. A plasmid construct which expresses Hck lacking a catalytic domain (pCAGGsHckN) in the pCAGGs mammalian expression vector was as described [45].

### 3.2. Antibodies and Cell Lines

The AG11, EH1 and AE6 mouse monoclonal antibodies to Nef were obtained from the NIH AIDS Research and Reference Reagent Program [83]. The mouse monoclonal antibody 19C was obtained from the Biomolecular Research Institute (Parkville, Australia). Pooled sera from HIV-positive human donors, biotinylated sheep anti-human IgG and Strepdavidin-HRP (Amersham, Little Chalfont, UK) were used to detect HIV proteins. Rabbit monoclonal anti-Hck (Santa Cruz Biotechnologies, Santa Cruz, CA, USA) and horseradish-conjugated mouse anti-rabbit IgG (Amersham, Little Chalfont, UK) was used for detection of Hck in all immunoblots. 293T cells were obtained from the American Type Culture Collection (ATCC) (Gaithersburg, MD, USA).

### 3.3. Viral Stocks

Viral stocks were generated by calcium-phosphate transfection of 293T cells with HIV-1 molecular clones. Luciferase reporter virus stocks were generated by co-transfecting 293T cells with the luciferase reporter construct and an envelope reporter construct in the presence and absence of Src kinase expression vectors. HIV-1-containing supernatant was harvested 48 h post-transfection, centrifuged to remove cellular debris and stored at −80 °C in 1 mL aliquots until required.

### 3.4. Infection of T Lymphocytes and Macrophages

Peripheral blood monocytic cells (PBMCs) were purified from healthy donor buffy coats (Red Cross Blood Bank, Melbourne, Australia) by Ficoll-paque (Axis-Shield, Oslo, Norway) isolation of leukocytes [84]. PBMCs were depleted of monocytes by adherence to plastic; non-adherent cells were collected as peripheral blood lymphocytes (PBL). Adherent monocytes were washed and collected. PBL were cultured in RF10 media [RPMI 1640 medium (Invitrogen, Carlsbad, CA, USA), 10% foetal calf serum, 100 U/mL penicillin/streptomycin and 60 mg/mL L-glutamine] at 2 × 10^6^ cells/mL. Monocytes were cultured at 1 × 10^6^ cells/mL in macrophage medium [Iscove’s media (Invitrogen, Carlsbad, CA, USA), 10% human sera (Sydney Blood Bank, Sydney, Australia or MP Biochemicals, Australia), 266 ng/mL gentamicin (Mayne Pharma, Melbourne, Australia) and 300 mg/mL L-glutamine]. PBL were infected on the day of isolation with 293T-derived viral stocks at 1 × 10^4^ reverse transcriptase (RT) units (virion-associated reverse transcriptase activity was measured using a micro-RT assay [85]) per 1 × 10^6^ cells and were stimulated with 10 µg/mL of phytohaemagglutinin (PHA; Murex, Dartford, UK) and 2 U/mL of interleukin 2 (IL-2, Roche Applied Science, Castle Hill, Australia) on the day of infection or 3 days post-infection. Monocyte-derived macrophages (MDMs) which had been allowed to mature for 5 to 7 days were infected with 1 × 10^5^ RT units of virus stock per 1 × 10^6^ cells.

### 3.5. Infectivity Assays

The relative infectivity of the HIV-1 infectious molecular clones AD8, AD8AxxA and AD8FSNef were determined by conducting a Tissue Culture Infectious Dose 50 (TCID_50_) assay in PBMCs and MDMs. Multiple wells (replicates of 6 wells per dilution) containing PBMCs or MDMs were inoculated with serial 1 in 10 dilutions of equivalent amounts of AD8 (Wt), AD8AxxA (Ax) or AD8FSNef (FS) based on virion-associated RT activity. At the peak of infection, supernatant from each well was assayed for RT activity as a measure of infection. TCID_50_ values were calculated using the method of Reed and Meunch [86]. MDMs and PBL were infected with luciferase reporter viruses generated in the presence and absence of wild-type and kinase-inactive Hck. MDMs were harvested 2 days post-infection. PBL were infected 3 days prior to, or 5 days after, activation with PHA and IL-2, and harvested 2 days post-activation. After harvesting, the cells were washed and lysed, and cell lysates standardized for protein content were subjected to a luciferase assay according to manufacturer’s instructions, using the SteadyGlo Luciferase Assay System (Promega, Fitchburg, WI, USA). Mock-infected cell lysates were used as negative controls and the signal generated from these was subtracted from the signal derived for each test sample to correct for background signal.

### 3.6. Immunoprecipitation Assays

Monocyte-derived macrophages infected with HIV-1 were harvested and lysed at day 7 post-infection. Lysates containing equivalent quantities of total protein were pre-cleared with Protein G Sepharose beads (Amersham Biosciences, Uppsala, Sweden) and pre-immune sheep sera (crude) for 30 min to 2 h at 4 °C with rotation. The cleared supernatant was incubated with 2 to 15 μL of affinity-purified Sheep 17 anti-Nef overnight at 4 °C with rotation. Protein G Sepharose beads were added to the mixture and incubated for 2 h at 4 °C with rotation. The beads were pelleted and washed 5 times in 1 mL of cold 1% Triton X-100 in PBS. After the final wash, the beads were incubated in 15–20 μL of 2% SDS in 0.5 M Tris-HCl (pH 8.0) for 30 min at 37 °C to elute the proteins. The supernatant was aspirated, added to 4 μL of SDS-PAGE loading buffer and heated at 100 °C for 2 min before being subjected to SDS-PAGE and transferred to nitrocellulose membrane. Western immunoblots were probed for the relevant Src kinase with rabbit anti-Hck at 1 in 1,000 dilution, and were also probed with rabbit anti-Nef.

### 3.7. Detection of Virion-Associated Nef and Src Kinases

Virus-containing supernatant harvested from transfected 293T cells or pooled from HIV-1 infected primary monocyte-derived macrophages (from ten donors per experiment) was pre-cleared of cellular debris by centrifugation at 3,000 ×*g* for 30 min at 4 °C and filtered through a 0.2 micron filter. Viral particles were purified by centrifugation and digestion with subtilisin for 16 hours at 37 °C as previously described [28,87]. Treated viral suspensions were diluted in 10 mL of PBS and centrifuged over a 20% sucrose cushion at 126,000 ×*g* for 1 h at 4 °C. The supernatant was decanted and the pelleted viral particles were lysed in 1% Triton X-100. Purified viral lysates were probed by Western immunoblot for the presence of Nef and Hck as described above, and for HIV protein with human sera (1 in 500 dilution), biotinylated anti-human IgG (1 in 1,000 dilution) and Strepdavidin-HRP (1 in 1,000 dilution). Prior to electrophoresis, the p24 content of each sample was were determined using the Vironostika p24 antigen ELISA kit (bioMerieux, Boxtel, The Netherlands) according to the manufacturer’s instructions, and equivalent quantities of p24 were analysed. Mouse monoclonal antibodies EH1 or AE6 (able to recognise the *C*-terminal 20 kDa fragment of Nef) were used to detect the 20 kDa cleaved form of Nef.

### 3.8. Statistical Analysis

Wilcoxon signed-rank tests were performed on the results of TCID50 infectivity assays in primary MDMs.

## 4. Conclusions

In summary, we have confirmed that the Src kinase Hck interacts with Nef during HIV-1 infection of primary monocyte-derived macrophages in a PxxP-dependent manner, and that both Nef and Hck are present in macrophage-derived virions. The incorporation of Hck into the virion is dependent on the presence of Nef, and is specifically dependent on the proline repeat motif of Nef. Hck inhibits infection of macrophages in a kinase-dependent manner, and this appears to be only partly dependent on the SH3 domain and therefore, potentially, incorporation of Hck into the virion, suggesting that Hck plays multiple roles in modulating viral infectivity. In contrast, Hck enhances the infection of primary T cells, suggesting that Nef interaction with Hck plays a role in enhancing the spread of HIV-1 from macrophages to other cell types.
